# Computational Insights on the Electrocatalytic Behavior of [Cp*Rh] Molecular Catalysts Immobilized on Graphene for Heterogeneous Hydrogen Evolution Reaction

**DOI:** 10.1038/s41598-020-62758-6

**Published:** 2020-04-01

**Authors:** Abdulilah Dawoud Bani-Yaseen, Elkhansa Elbashier

**Affiliations:** Department of Chemistry & Earth Sciences, College of Arts & Science, Qatar University, P.O. Box 2713, Doha, State of Qatar

**Keywords:** Computational chemistry, Electrocatalysis

## Abstract

The heterogeneous metal-based molecular electrocatalyst can typically exhibit attractive features compared to its homogeneous analogue including recoverability and durability. As such, it is necessary to evaluate the electrocatalytic behavior of heterogenized molecular catalysts of interest toward gaining insights concerning the retainability of such behaviors while benefiting from heterogenization. In this work, we examined computationally the electrochemical properties of nanographene-based heterogenized molecular complexes of Rhodium. We assessed, as well, the electrocatalytic behavior of the heterogenized molecular catalyst for hydrogen evolution reaction (HER). Two electrochemical pathways were examined, namely one- and two-electron electrochemical reduction pathways. Interestingly, it is computationally demonstrated that [Rh^III^(Cp*)(phen)Cl]^+^-Gr can exhibit redox and electrocatalytic properties for HER that are comparable to its homogeneous analogue via a two-electron reduction pathway. On the other hand, the one-electron reduction pathway is notably found to be less favorable kinetically and thermodynamically. Furthermore, molecular insights are provided with respect to the HER employing molecular orbitals analyses and mechanistic aspects. Importantly, our findings may provide insights toward designing more efficient graphene-based molecular heterogeneous electrocatalysts for more efficient energy production.

## Introduction

Molecular electrocatalysts have recently gained great level of interests due to its practicality in promoting various chemical transformations via redox mediation for energy applications^1–5^. In principle, these electrocatalysts are generally synthetic in nature that comprise transition metals coordinated with a variety of ligands. Commonly, these electrocatalysts are synthetically prepared and tested as homogeneous molecular electrocatalysts for various types of important chemical transformations of interest, such as the hydrogen evolution reaction (HER)^4,6–10^. Prospectively, the heterogeneous versions of such electrocatalyst can be advantageous in terms of robusticity and practicality toward their potential integration within devices for redox-mediated energy conversion. Hence, great interests have recently grown toward such heterogenization of molecular electrocatalysts using various types of nanomaterial, such as carbon nanotubes and graphene^11–14^.

Efforts concerning heterogenization of molecular electrocatalysts have focused on the attachment of the homogeneous molecular catalysts to the surface of nanomaterials serving as electrodes for redox mediation catalysis of reactions of interests. Various approaches have been developed in this regards, this includes self-assembled monolayers (SAMs), or via noncovalent immobilization such as π-π stacking^11–17^. However, for the heterogenized molecular electrocatalysts, it must be mentioned that efficient electronic coupling between the electrode and molecular catalysts is crucial toward retaining the characteristics electrocatalytic properties of the homogeneous analogue while functioning as heterogeneous electrocatalysts. Notably, recent approaches have rationalized the covalent attachment of the molecular homogeneous catalysts to the surface of electrochemically active carbon materials. For example, Surendranath *et al*. have reported on the immobilized heterogeneous catalyst of Rh metal complexes on graphite-conjugated catalyst linked via pyrazine groups^18^. In their work, they demonstrated experimentally that the homogeneous molecular catalysts [Rh^III^(cp*)(bpy)Cl]^1+^ can exhibit different redox behavior compared to the heterogenized analogue in acetonitrile as they undergo two-electron metal-centered and one-electron non-metal-centered reduction pathways, respectively. However, although many studies have been reported since the early work of Grätzel and Kölle concerning the catalytic properties of the homogeneous analogues of Rh complexes^19–26^, no results have been yet reported concerning the assessments of the catalytic behaviors of the heterogenized molecular catalysts for the HER.

**Figure 1:**
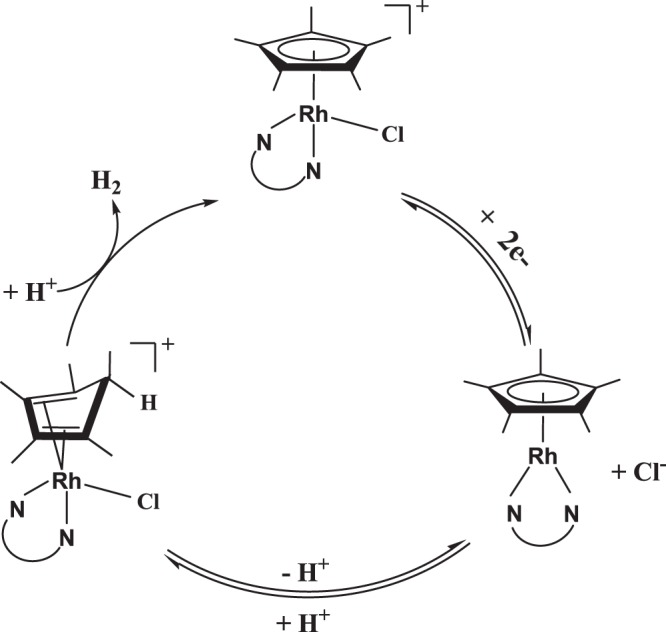
Catalytic cycle for H_2_ evolution catalyzed by Rh(Cp*)(L).

Importantly, as mentioned above, the cruciality of efficient electronic coupling to the electrode necessitates evaluating the redox behavior of such molecular electrocatalysts before and after heterogenization and consequently evaluating their catalytic behavior toward a chemical transformation of interest, such as the HER. On the other hand, the applicability of Rh(Cp*)-based homogeneous molecular catalysts in acetonitrile has recently been interestingly addressed toward electrochemical hydrogen generation. The corresponding catalytic cycle (Fig. 1) comprises the formation of isolable intermediate of Rh complex that bears [η^4^-Cp*H] as a ligand^27–29^.

**Figure 2:**
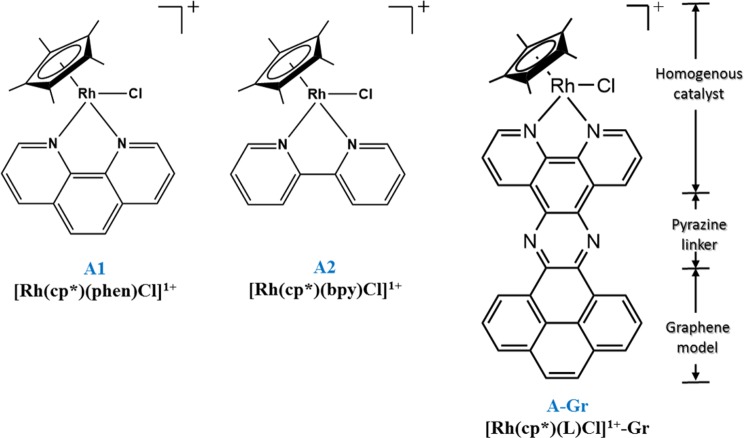
Chemical structure of redox species considered in this study.

Importantly, there is a growing necessity toward providing computational-based molecular interpretations and insights concerning the electrocatalytic properties of the molecular heterogeneous electrocatalysts compared to its homogeneous analogue, which in turn is significant for improving their performance towards developing electrocatalyst by design^30–34^. In the light of the importance of heterogenization of transition metal-based molecular electrocatalysts and inspired by the recent work of Surendranath *et al*., we aimed in this work using Density Functional Theory (DFT) to computationally evaluate the redox behavior of selected graphene-based heterogenized molecular Rh complexes ([Rh^III^(Cp*)(L)Cl]^1+^) and the corresponding electrocatalytic activity for the HER. Both behaviors of interest, namely redox and electrocatalysis of the HER, are comparatively evaluated for both phases at the molecular levels. The assessment includes selectively redox active species as displayed in Fig. 2. These species were selected based with respect to the catalytic cycle shown in Fig. 1. Interestingly, the findings of this study may help in developing new heterogenized molecular catalysts or enhancing the electrocatalytic performance of existing ones.

## Computational Methods

All calculations were conducted using Gaussian09 software package, Revision E.01^35^. Geometry optimization of all complexes were performed using DFT method with B3LYP functional followed by frequency calculations. 6–31 G(d) basis set was used for the non-metals, C, H, N, and Cl atoms, whereas lanl2dz for Rh. The graphene sheet (Gr) was modelled as four fused benzene. Selected pentamethylcyclopentadiene-based Rhodium complexes [Rh^III^(Cp*)] are considered in this work. Figure 2 illustrates the chemical structure of complexes considered in this work and the corresponding homogeneous analogues, namely [Rh^III^(Cp*)(L)Cl]^1+^ (L = (2,2′-bipyridyl) (bpy) or phenolphthalein (phen). For the calculations of the solvent effects, an implicit solvation method of the integral equation formalism variant of the Polarizable Continuum Model (IEFPCM) was used in acetonitrile (ε = 35.688).

### Redox potential calculations (${{\bf{E}}}_{{\bf{red}}}^{{\bf{o}}}$)

**Figure 3:**
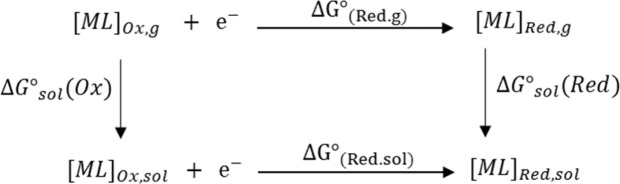
Born−Haber cycle for the computation of reduction potential of [ML]^n+^ in solution.

The ${{\rm{E}}}_{{\rm{red}}}^{{\rm{o}}}$ of molecules of interest was calculated employing the Born–Haber cycle as illustrated in Fig. 3 using the DFT optimized geometry of molecule of interest in the corresponding medium^36,37^. The change in standard Gibbs free energy of the reduction of the corresponding substance in solution is (∆G^o^_(Red,sol)_) and the corresponding ${{\rm{E}}}_{{\rm{red}}}^{o}$, vs. Ferrocenium/ Ferrocene (E^o^ (Fc^+/0^))^38^, were calculated using Eqs. 1 and 2, respectively.

1 $$\Delta {G}_{(Red,sol)}^{o}=\Delta {G}_{(Red,g)}^{o}+\Delta {G}_{sol}^{o}(Red)-\Delta {G}_{sol}^{o}(Ox)$$

2 $${E}^{^\circ }(V)=\frac{-\Delta G{^\circ }_{(Red.sol)}}{nF}-E^\circ (F{c}^{1+/0})$$

where, n is the number of electrons involved in the half-reaction, and F is the Faraday constant.

## Results and Discussion

The analyses of interest here are focused on insighting the redox and the corresponding electrocatalytic behaviors of the metal-based heterogenized molecular catalysts of selected Rhodium complexes compared to the homogenous analogue in terms of structural properties, mechanistic aspects, and molecular orbitals. It must be mentioned that performing such evaluating protocols requires initially performing accurate DFT-based geometry optimizations for all species that are involved in the electrochemical reduction and catalytic cycle of interest. According to previously reported experimental results concerning the redox behavior of complexes of [Rh(Cp*)(L)Cl]^+^ (L= bpy or phen), such complexes commonly undergo a metal-centered two-electron reduction (Rh^III/I^) at approximately −1.19 V vs Fc^+/0^,with simultaneous dissociation of chloride ion (Cl^−^)^18,21,22^; whereas the heterogenized analogue undergoes a nonmetal-centered one-electron reduction at −1.29 V vs Fc^+/0^ with alike dissociation of Cl^18^. As such, we optimized the geometries of these species that are involved in the electrochemical reduction using DFT calculations. Coordinates of the optimized geometries in acetonitrile are provided in the supplementary information.

**Figure 4:**
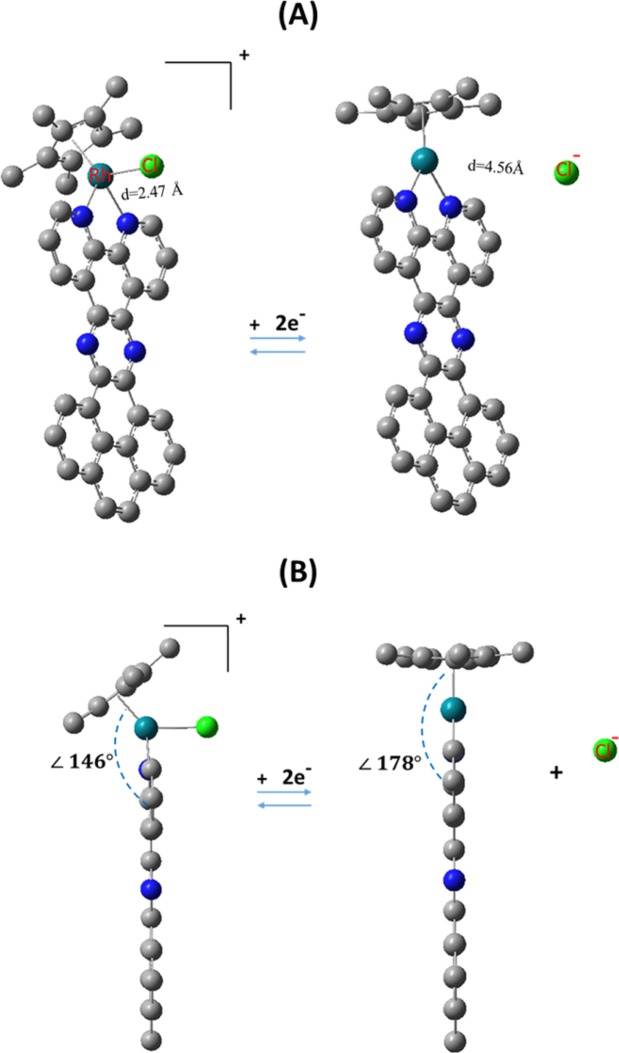
Front (**A**) and side (**B**) views of optimized geometries of A-Gr before and after electrochemical reduction in acetonitrile; hydrogen atoms were omitted for clarity.

Figure 4A displays the corresponding optimized geometries before and after electrochemical reduction of the heterogenized molecular catalysts (A-Gr). As can be noticed, as in good agreement with the reported experimental results, the reduction process has induced an increase in the length of Rh-Cl bond from 2.47 Å to 5.39 and 4.56 Å for the homogeneous and analogous heterogenized catalysts, respectively, indicative of full bond breakage and detachment of Cl^−^. Notably, such breakage of the Rh-Cl bond was obtained for both processes of two-electron and one-electron electrochemical reduction. On the other hand, the DFT based calculated geometrical properties were benchmarked with the reported XRD crystallographic data by Blakemore *et al*. for analogous complexes; particularly, [Rh^III^(Cp*)(bpy)X]^+^ (X:Br, Cl)^39,40^. We emphasized herein on the geometrical properties after the electrochemical reduction process. It must be mentioned that the experimental data was reported for the Bromide ligand, whereas the calculation here was performed for the Chloride ligand. However, in both cases, the product of the electrochemical reduction must have the same structure as of ejecting the halogen ion ligand. For example, bond lengths for Rh-C(Cp*) in the range of 2.211–2.253 Å was obtained in vacuum and acetonitrile for [Rh^III^(Cp*)(bpy)]^2+^ after the two-electron reduction process, which are in good agreement with the corresponding experimental values of 2.190–2.223 Å. For the heterogeneous analogues, Rh-C(Cp*) in the ranges 2.239–2.295 and 2.264–2.330 Å, were obtained after the one-electron and two-electron reductions processes in vacuum, respectively, indicative of ignorable change in the corresponding bond length after heterogenization and independent of the number of electrons transferred. Furthermore, similar behaviors were obtained for the C-C bonds length within the Cp* and bpy ligands. Furthermore, a notable change in the geometry of the reduced forms in both phases is the dihedral angle between the plane of phen containing Rh and the Cp* ring; see Fig. 4B. For example, a change in the angle from 146 to 178° was computed, which is in good agreement with 179° obtained for [Rh^III^(Cp*)(bpy)]^2+^ after electrochemical reduction^39,40^.

Importantly, it is noteworthy to mention that this electrochemically induced dramatic change in the geometrical properties of the electrocatalyst might be crucial to initiate the binding to substrate of interest that in turn to be catalytically converted, such as the HER. In addition, as mentioned in the methodology section, performing the DFT calculations in different solvents is essential for obtaining the ∆G^o^ of solvation needed for calculating the redox potential in the corresponding medium (infra vide). Indeed, the DFT calculations revealed a negligible effect of solvation (acetonitrile) on the structural properties of the examined systems, this includes the main bonds length and angles of atoms that can be potentially involved directly in the catalytic and electrochemical behavior of the molecule.

### Calculated Reduction Potentials

It is noteworthy mentioning that the electrochemical assessment of a potential electrocatalysts is crucial toward developing efficient electrocatalysts of interest of relatively low overpotential, which in turn is highly desired for electrocatalytic production of H_2_. As such, the thermodynamics quantities obtained from the DFT calculations, single-point frequency calculation of the optimized geometry in particular, were utilized for calculating the change in free energy of each step illustrated in Fig. 3. These are accordingly needed for calculating the electrochemical ${{\rm{E}}}_{{\rm{red}}}^{{\rm{o}}}$ of all systems considered in this study. Importantly, as it has been demonstrated computationally by several research groups, it must be mentioned that there is no universal DFT functional that can accurately predict the ${{\rm{E}}}_{{\rm{red}}}^{{\rm{o}}}$ for all substances in different media^36,37^. As some functionals perform very well for such prediction for some substances, they totally fail for others based on benchmarking against experimental values. For example, as recently reported by Liang *et al*.^36^, B3LYP functional with a combination of LANL2DZ and 6–31 G(d) basis set for metal and non-metal atoms, respectively, and IEFPCM implicit solvation model, perform quite-well for wide range of organometallic substances with mean absolute deviation (MAD) of 0.233 V; yet, this method fails for some other substances. Nevertheless, considering the main two factors in computational chemistry, namely accuracy and computational cost, this method was highly recommended^36^. To this end, the ${{\rm{E}}}_{{\rm{red}}}^{{\rm{o}}}$ of molecules of interest was calculated using the Born-Haber thermodynamic cycle with optimized geometries of all molecules simulated employing the recommended level of theory mentioned above. Our first attempt was to perform the calculations for species that can be benchmarked with experimental values. Hence, the calculations were conducted for the ${{\rm{E}}}_{{\rm{red}}}^{{\rm{o}}}$ (vs. Fc^+/0^) of the molecular reduction models of two-electron and one-electron for the homogeneous and heterogenized analogue, respectively. However, further calculation were conducted for the presumable 2-electron reduction of the heterogenized electrocatalyst. Importantly, the validity of the calculation method applied in this work is evaluated through benchmarking with reported experimental values wherever applicable. As such, these theoretical values were calculated versus the appropriate reference electrode as indicated by the reported experimental results, namely Fc^+/0^ ^18,21,36^.

It is noteworthy to mention that the key point herein is to examine the effect of heterogenization on the redox potential of the Rh molecular electrocatalyst. All results are compiled in Table 1. As can be noted, the DFT calculations afforded ${{\rm{E}}}_{{\rm{red}}}^{{\rm{o}}}$ of −1.07 and −1.05 V for complexes A1 and A2, respectively, which are in good agreement with the experimental values of −1.19 and −1.21 V, respectively^18,21,36^. However, a notable discrepancy for the calculated ${{\rm{E}}}_{{\rm{red}}}^{{\rm{o}}}$ of the heterogenized analogous is observed. This can be attributed to various reasons that could be either computational or even experimental such as scanning rate and type of electrodes and concentration of the supporting electrolytes. Nevertheless, the majority of halide-bound diimine complexes if [Cp*Rh^III^] can generally undergo two-electron electrochemical reduction that is observed experimentally as a single process^18,21,22,36,41^. Further attempts were conducted to reduce the discrepancy between the calculated and the experimental value of the 1-electron reduction process. In this regard, we increased the number of the benzene fused rings to seven and we examined the effect of another DFT functionals, namely M06; see Fig. 1S. However, none of these attempts has significant effect on the calculated ${{\rm{E}}}_{{\rm{red}}}^{{\rm{o}}}$. In the light of high E°red (vs. Fc^+/0^) calculated for the one-electron reduction of A-Gr. Adding more fused rings has shown no improvement on the ${E}_{{\rm{red}}}^{o}$ of the one-electron process and hence no further attempts were conducted for including the seven-ring systems in our results. Thus, we extended our investigation into calculating the ${{\rm{E}}}_{{\rm{red}}}^{{\rm{o}}}$ of presumable 2-electron reduction of A-Gr^+/−^. As noted in Table 1, the DFT calculations for the presumable two-electron reduction of A-Gr^+/−^ afforded a ${{\rm{E}}}_{{\rm{red}}}^{{\rm{o}}}$ of −1.05 V vs Fc^+/0^ that is more comparable with the experimental values of the homogeneous analogue of various members of the [Cp*Rh^III^] family.

**Table 1:** Reduction potential (V) vs. Fc^+/0^ of redox active species considered in this study.

${{\bf{E}}}_{{\bf{red}}}^{{\bf{o}}}$	Redox Complex			
	A1^+/−^	A2^+/−^	A-Gr^+/0^	A-Gr^+/−^
Calc.	−1.07	−1.05	−2.11	−1.05
Expt.^#^	−1.19	−1.21	−1.29	—

#Ref. ^18,21,22^.

These DFT results indicate that the electrochemical molecular properties of the species considered in this study are slightly dependent on the types of the diamine ligands, namely phen and bpy. This, in turn in good agreement with the recently reported results for such effects^22^. For selected Cp*Rh complexes, Henke *et al*. reported ${{\rm{E}}}_{{\rm{red}}}^{{\rm{o}}}$ (vs. Fc^+/0^ in acetonitrile) of −1.25 V for the 4 and 4′ t-butyl disubstituted phen ligand compared with −1.21 V for the unsubstituted one. However, notable decrease in the ${{\rm{E}}}_{{\rm{red}}}^{{\rm{o}}}$ to −0.97 V was recorded for the –CF_3_ substituents at the same positions of substitution. Nevertheless, it is highly anticipated that extending the range of such substituents may help in tuning the n-electron reduction (n:1 ↔ 2) of the heterogenized analogue of the molecular electrocatalysts^22–24^.

**Figure 5:**
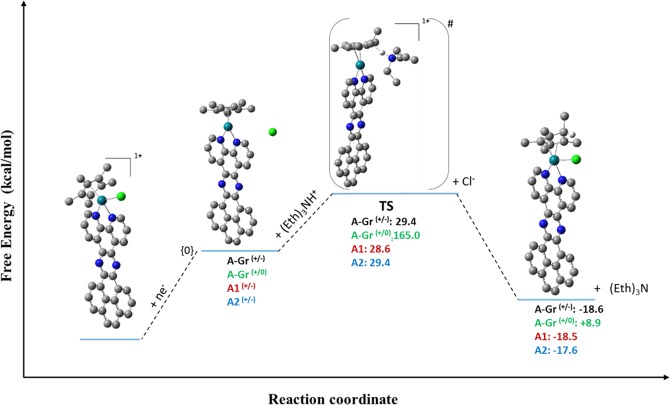
Free energy profile (with respect to the reduced specie) for the electrochemical reduction and the consequent protonation of the [η^5^-Cp*] ligand to form [η^4^-Cp*H] of the redox active species considered in this study.

Importantly, the significance of heterogenization of a molecular electrocatalyst can be generally assessed through investigating their catalytic performance toward a reaction of interest, such as the HER; this includes evaluating the role of n-electron reduction. As illustrated in Fig. 4, Rh complex bears [η^4^-Cp*H] is the main intermediate that is formed during the catalytic cycle of HER; yet, other intermediates can still be potentially formed including the metal-hydride species^25–29,42^. In the work of Johnson *et al*.^29^, they examined using DFT with M06 functionals the mechanism of HER using homogeneous molecular catalysts of Cp*Rh(bpy). Still, in our work we utilized different functional for studying the process of interest the decisive approach to be followed is based on the benchmarked analysis of the calculated reduction potential of the molecules of interest. Indeed, the approach that we utilized afforded relatively better performance concerning the electrochemical properties, and hence, for the purpose of consistency, we employed the same approach for investigating the underlying mechanism of the HER suing the homogeneous molecular catalysts of Cp*Rh(bpy) and their graphene-heterogenized analogues. Decisively, as the primary focus of this work is to consider a comparative approach concerning the homogeneous molecular catalyst of Rh versus its heterogenized analogue toward the catalyzed HER, we focused on examining the non-innocent role of [Rh-η^5^-Cp*] ligand and the corresponding transformation to its protonated analogue [Rh-η^4^-Cp*H] as the key transformational step within the catalytic cycle of the HER that is in turn essential for initiating the process. Hence, the objective of this approach is to provide quantitative thermodynamic and kinetic criteria toward comparative evaluation of a heterogenized molecular catalyst versus its corresponding analogue. On the other hand, protonation of the Cp* ligand was modeled explicitly using a weak acid, namely Et_3_NH^+^. As such, the energy diagram obtained by free energies differences for such catalytic cycle comprising the species of interest is displayed in Fig. 5.

In Fig. 5, the calculated values for each specie is considered as relative to the electrochemical reduced form of the corresponding catalysts, where the change in Gibbs free energy (∆G) of the reduced form of the catalyst is set to zero. In this regard, both ∆G^#^ and ∆G° were assessed, which refer to the thermodynamics related to the formation of the excited state and the products, respectively. As it can be noticed from the energy profile, A1 and A2 exhibited approximately an identical catalytic behavior kinetically and thermodynamically with a negligible difference in ∆G^#^ and ∆G° of only 0.8 and 0.9 kcal/mol, respectively. Likewise for the two-electron reduction pathway of A-Gr^+/−^ compared to the homogeneous analogue of A1 and A2, where almost the same typical behavior is repeated. For example, the DFT calculations afforded ∆G^#^ and ∆G° of −29.4 and −18.6 kcal/mol, respectively, indicating a feasible kinetic barrier and spontaneous process for forming the protonated ligand complex [η^4^-Cp*H], which in turn facilitating expectedly the imitation process of the HER. However, as revealed by the DFT calculations, it is evident that the formation of [Rh^I^ (η^4^-Cp*H)]-Gr through a two-electron reduction process is kinetically and thermodynamically favored over the one-electron reduction process to form [Rh^II^ (η^4^-Cp*H)]. Other important factor to be mentioned herein is the solvent effect. Notably, the implicit solvation effect has increased the activation energy of the protonation step from 7.2 kcal/ mol in vacuum for A-Gr^+/−^ into 29.4 kcal/mol in acetonitrile. Alike solvation behavior was observed for all examined species. Furthermore, another issue that is worth to be addressed structurally is the bonds’ length of atoms that are most likely to be involved in the catalytic process of the HER. A1, A2, and A-Gr^+/−^ exhibited similar structural properties including those of the main bonds involving Rh. For example, for the protonated complexes of A, A2, and A-Gr^+/−^ in acetonitrile, almost identical bond length of 2.160 (for all four carbon atoms of (η^4^-Cp*H)) and 2.680 Å were calculated for Rh- C_Cp*_ and Rh- C_Cp*_(H), respectively. However, although a bond length of 2.80 Å was calculated for Rh- C_Cp*_(H) of A-Gr^+/0^, two distinctive bonds length was observed for the other four carbon atoms of (η^4^-Cp*H) with an average of 2.374 and 2.253 Å.

### Molecular orbitals

**Figure 6:**
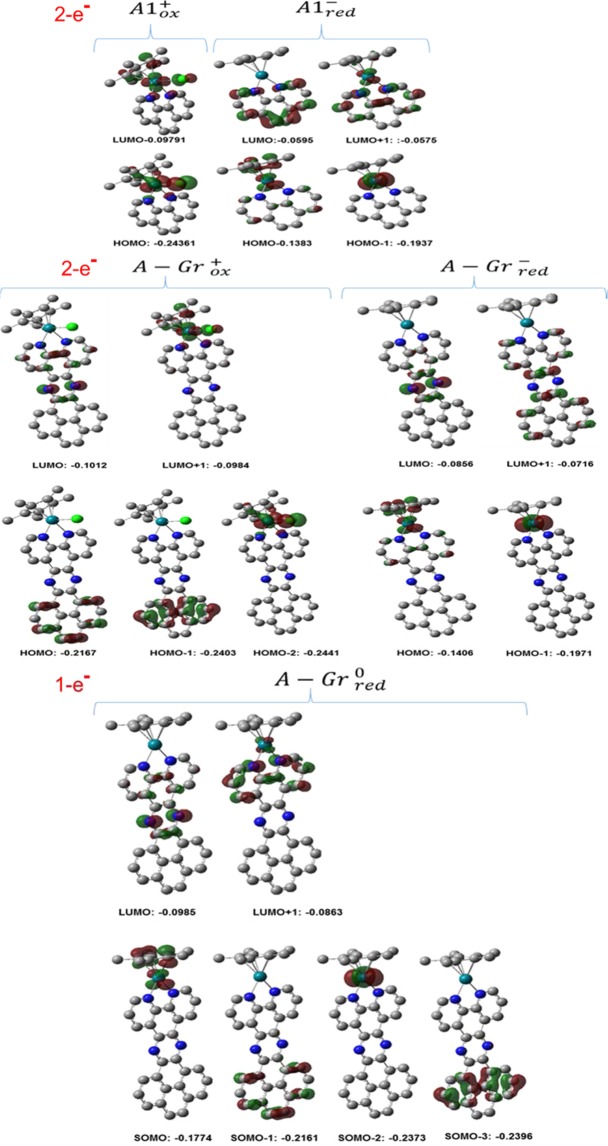
MOs of A1 (2-e^−^) and A-Gr before and after 2-e^−^ and 1-e^−^ electrochemical reduction; energy in eV.

To provide further insights at the molecular level concerning the catalytic behaviors toward the HER of the species considered in this study, we performed molecular orbitals analyses. It is noteworthy mentioning that providing insights regarding the properties of the frontier molecular orbitals, namely highest occupied molecular orbital (HOMO) and lowest unoccupied molecular orbital (LUMO), of a catalysts is important toward enhancing the understanding of the corresponding electrochemical catalytic behavior. In principle, the latter is significantly important in electrochemical catalyzed reduction of substances of interest, such as H_2_, where a lower energy for the LUMO can desirably assure lower electrochemical overpotential without significantly affecting the overall catalytic efficiency. As such, we conducted a simulation for the MOs of interest including the frontier ones for all complexes considered in this study. Hence, one approach of analyses is visualizing the MOs of the heterogenized molecular catalyst and compare it with the MOs of its corresponding homogeneous analogue. Selected MOs of all [Rh(Cp*)(phen)Cl]^1+^ before and after heterogenization are displayed in Fig. 6.

It is indeed important to consider the localization pattern and energies of the MOs and their corresponding changes during the catalytic reaction for both transformations, namely before and after heterogenization, as well as the influence of electrochemical reduction. Selected MOs of A1^+/−^, one-electron reduction A-Gr^+/0^, and two-electron reduction A-Gr^+/−^ are displayed in Fig. 6. Before reduction, the frontier MOs of the homogeneous catalyst A1 are delocalized over the metal and the surrounding ligands Cl, Cp*, and nitrogens of phen. Upon reduction and release of Cl^−^, it is expected that the additional two electrons of reduction will be placed in the LUMO to afford new HOMO with calculated stabilization of 0.0404 eV from −0.0979 to −0.1383 eV. This new HOMO as can be noticed in Fig. 6 is localized over the metal and important unignorable delocalization over the Cp* ligand. As in good correspondence with the proposed mechanistic cycle, this makes both of them, the metal and Cp*, more likely to be involved during the process of the HER indicative of the non-innocence role of the ligand. Obviously, affording new HOMO has led to the formation of new LUMO and LUMO+1 that are of energy of −0.0595 and −0.0575 eV, respectively, which are delocalized mainly over the phen ligand, which on the other hand most likely has no significant contribution within the catalytic cycle of the HER. However, HOMO-1 is a dz^2^ metal orbital with no interaction with the surrounded ligands with an energy of −0.1937eV. However, in correspondence with the metal centered two-electron reduction process, it must be mentioned that such process may have shifted the relative energy of the MOs to place the dz^2^ metal orbital of Rh in the position of HOMO-1 as revealed by the DFT calculations. Comparing this behavior with the heterogenized analogue, it can be noticed that after heterogenization the positions of the LUMO and HOMO have changed with the addition of new MO from the conjugated system of the pyrazine linker and graphene. The LUMO has shifted to LUMO+1 with an energy of −0.98 eV and HOMO has shifted to HOMO-2 with an energy of −0.244 eV, which indeed is more stable compared to the HOMO of the homogenous analogue. Nevertheless, the two-electron reduction has interestingly afforded a new HOMO of the same shape of comparable energy as of the homogeneous analogue. This new HOMO in fact is produced from the LUMO+1 rather than the LUMO. Importantly, this new HOMO, regardless of its source, may expectedly processed with the same catalytic behavior for the HER, which in turn is consistent with the similarities in the ${{\rm{E}}}_{{\rm{red}}}^{{\rm{o}}}$ observed for both catalysts. For the one-electron reduction, alike behavior is observed in terms of the place where the new electron to be located; i.e. the new electron was added to the LUMO+1 to form the SOMO. However, one can notice that the stabilization energy is 0.0790 eV compared with 0.0404 eV for the HOMO of the reduced form of the two-electron reduction of A-Gr. This in fact is in good correspondence with the large difference in activation energy observed for forming the transition state of the protonated moieties, where more stabilized HOMO necessitates higher activation energy in consequent transformations. Indeed, this explains as well the high value obtained for the one-electron reduction of A-Gr compared with the two-electron reduction. Importantly, examining the nature of the generated SOMO of the one-electron reduced catalysts, one can notice that it is distributed mainly over the metal with considerable delocalization over the Cp* ligand indicative of potential non-innocent role for the ligand.

**Figure 7:**
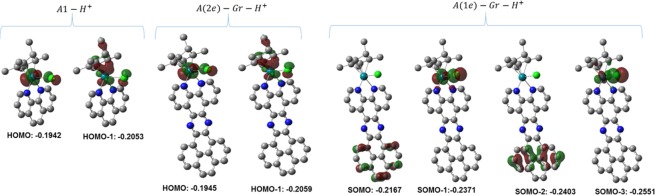
Occupied MOs of A1 and A-Gr after protonation; energy in eV.

Furthermore, The MOs before and after protonation accompanied by the re-coordination with the Cl^−^ were analyzed. It is noteworthy to mention that the addition of Cl^−^ will relatively affect the energy of the MOs, and hence the comparison herein is based on qualitative aspects. Also, the MOs that are considered herein are the occupied ones that may exhibit major contribution in the catalyzed HER. Selected occupied MOs of A1^+/−^, one-electron reduction A-Gr^+/0^, two-electron reduction A-Gr^+/−^ after protonation are displayed in Fig. 7.

As shown in Fig. 7, the HOMO of the reduced forms of A1 and A-Gr (2e) comprises not only a metallic features, but Cp* features as well, permitting for attracting H^+^ to form the protonated species. For the protonated species of the homogeneous catalysts and its heterogenized analogue (2e), and considering the re-coordination of Rh with Cl^−^, a new HOMO is formed that is localized on the metal and chloride ligand indicating that further protonation will most likely be a metallic-centered process, which in turn can be consequently followed by evolution of H_2_ and regenerating the original catalyst. However, the protonated 1e specie of the heterogenized catalyst (A(1e)-Gr-H^+^) exhibited a different behavior, where the SOMO is localized on the graphene ligand. This new SOMO in turn may shield the SOMO-1 orbital, which is of the same characters as that of the HOMO of the A(2e)-Gr-H^+^, and hence may retard further processes for the HER. In fact, this non-metallic centered SOMO is more likely consistent with the non-metallic electrochemical one-electron reduction of the heterogenized catalyst that was observed experimentally^18^.

## Conclusions

We have demonstrated computationally using DFT approach that the nanographene-pyrazines-based covalently heterogenized molecular catalysts of [Rh^III^(Cp*)(phen)Cl]^+^ can exhibit typical two-electron redox behavior and electrocatalytic properties for the HER that are comparable to its behavior as homogeneous electrocatalyst. Importantly, the appropriateness of the DFT-based redox calculations employing Born–Haber cycle in combination with IEFPCM solvation model in acetonitrile is benchmarked through comparison with alike experimental systems that were previously reported. The appropriate modelling of nanographene sheet was simulated and successfully demonstrated through implementation of a four-fused rings, where it is demonstrated that adding more rings to the model did not enhance the accuracy of the calculations. Furthermore, it is demonstrated through the DFT calculations that the two-electron reduction pathway can afford more favorable kinetic and thermodynamic pathway for the HER compared with the one-electron pathway, which in turn is in good correspondence with the behavior of the homogeneous analogue. However, with the high value obtained for the one-electron reduction potential of the heterogeneous catalysts, it sounds challenging to utilize such graphene-based covalently heterogenized molecular catalysts of Cp*Rh for experimental H_2_ evolution. Interestingly, the findings reported herein may aspiringly be used for further designing of novel and more efficient heterogeneous molecular catalysts.

## Supplementary information

Supplementary Information.
