# Supplementary material for: Computational Insights on the Electrocatalytic Behavior of [Cp*Rh] Molecular Catalysts Immobilized on Graphene for Heterogeneous Hydrogen Evolution Reaction
Source: Sci Rep. 2020 Apr 1;10:5777. doi: 10.1038/s41598-020-62758-6 (PMC7113254; doi:10.1038/s41598-020-62758-6)
Supplement: Supplementary file 1 — Supplementary Information. [file 41598_2020_62758_MOESM1_ESM.docx]

**Computational Insights on the Electrocatalytic Behavior of [Cp*Rh] Molecular Catalysts Immobilized on Graphene for Heterogeneous Hydrogen Evolution Reaction**

Abdulilah Dawoud Bani-Yaseen^*^, Elkhansa Elbashier,

Department of Chemistry & Earth Sciences, College of Arts & Science, Qatar University, Doha, P.O. Box 2713, State of Qatar

**
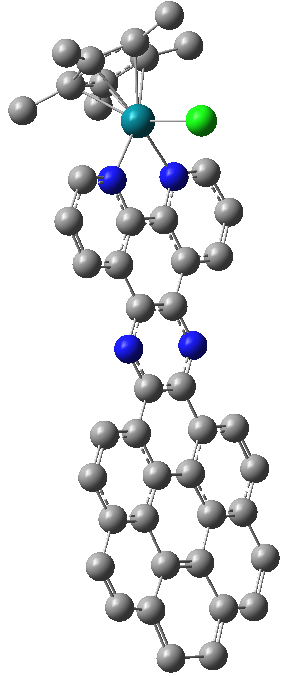
**

Figure 1S. The seven-ring model of the graphene surface of [Rh^III^(Cp*)Cl]^1+^-G_C_; hydrogen atoms were omitted for clarity

**Computational Details**

*All calculations were conducted using Gaussian09 software package, Revision E.01 ^1^*

Cartesian coordinates (in Å) and total energies (in a.u.) of optimized geometries in acetonitrile using DFT method in combination with IEFPCM solvation model.

| **A1^1+^** | |
| --- | --- |
| Energy | -1531.38840745 |

**
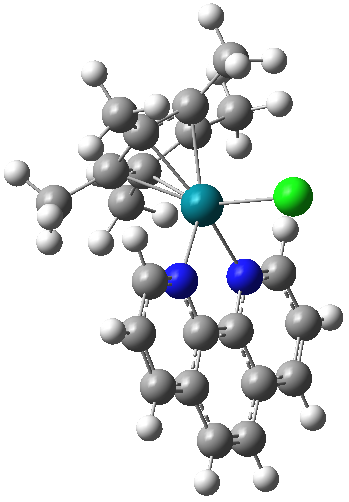
**

C 1.25673400 5.26341900 -0.14869200

C 2.60778900 5.00902400 -0.28349500

C 3.52480300 6.08197900 -0.22666200

C 2.99537300 7.38215700 -0.04788600

C 0.81827200 6.58716700 0.02968200

C 4.94759600 5.91865500 -0.32924300

C 3.87631200 8.50666200 0.05024100

C 5.27602600 8.31640500 -0.03455000

C 5.78696300 6.98980800 -0.23751500

C 6.09588800 9.45909800 0.09830500

H 7.17501700 9.35718100 0.03580400

C 5.51126700 10.69131300 0.31708100

C 4.11047200 10.78762200 0.39270500

H 0.52527600 4.46383800 -0.17568100

H 2.97310100 3.99627000 -0.42337500

H -0.23464400 6.80987600 0.15270500

H 6.10934900 11.58786900 0.43356300

H 3.63105100 11.74141800 0.57668200

N 3.31479100 9.73056600 0.25066400

N 1.65959300 7.61767900 0.06435700

Cl 1.36103700 9.31098400 2.75379500

Rh 1.15668800 9.69332400 0.32330700

C -0.21763900 9.93583700 -1.41822500

C -0.58290000 11.10392600 0.56882500

C 0.47715900 11.76182300 -0.13856400

C -0.98543400 9.94549200 -0.18602800

C 0.69107400 11.04276100 -1.38497800

C -1.19248100 11.55555100 1.85464200

H -0.49983100 12.16135200 2.44184900

H -2.07440100 12.17058600 1.62941900

H -1.51712100 10.71065400 2.46522300

C -2.13853200 9.04947200 0.14914400

H -3.07914500 9.52862600 -0.15215900

H -2.08097000 8.09332000 -0.37699900

H -2.19583500 8.85064800 1.22252100

C 1.10577200 13.06575300 0.24671400

H 2.07315900 13.21358300 -0.23918600

H 0.45708500 13.89365100 -0.06769900

H 1.24553500 13.14623400 1.32783900

C 1.62933700 11.45174100 -2.47633900

H 1.94409500 10.59866400 -3.08178600

H 1.12739800 12.16837800 -3.13929200

H 2.52352200 11.94021000 -2.08123600

C -0.39968300 8.97873000 -2.55305800

H -0.68903600 7.98294400 -2.20839000

H -1.20167000 9.34509500 -3.20713500

H 0.50660400 8.88521600 -3.15516900

H 6.86189400 6.85653200 -0.30891200

H 5.34255500 4.91798300 -0.47433200

| **A2^1+^** | |
| --- | --- |
| Energy | -1455.15688322 |

**
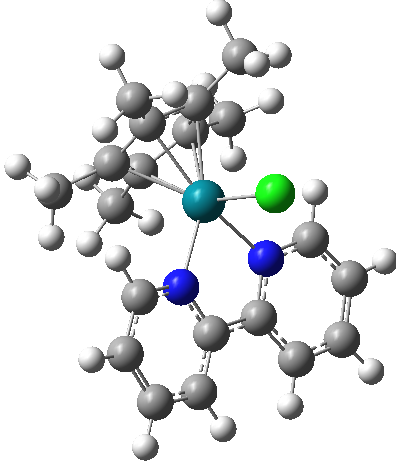
**

C 1.50703300 5.32897200 -0.90322400

C 2.88839500 5.15069500 -0.94239900

C 3.71782100 6.21583600 -0.60283600

C 3.15248900 7.44299700 -0.24476200

C 1.00700200 6.57689000 -0.54449900

C 3.93869900 8.60838100 0.20022800

C 5.33232700 8.60967900 0.30900500

C 5.97997000 9.73685100 0.80730500

H 7.06119300 9.74901400 0.89506600

C 5.21962200 10.83573900 1.20192900

C 3.83640200 10.77275400 1.06263400

H 0.82098900 4.52481300 -1.14301800

H 3.31769300 4.19452100 -1.22231500

H -0.05847100 6.75829600 -0.49288400

H 5.67777600 11.72869500 1.61140000

H 3.20567500 11.59895300 1.36358600

N 3.21380700 9.69726700 0.55831700

N 1.80639700 7.61085800 -0.24110600

Cl 0.94746400 8.74119800 2.63176400

Rh 1.09898700 9.54767100 0.28917000

C -0.49409100 9.79646100 -1.28852600

C -0.37352400 11.22024900 0.55680100

C 0.60400900 11.64910200 -0.38620300

C -1.05187700 10.05152300 0.00355100

C 0.58741300 10.72946700 -1.50927200

C -0.74517300 11.88924700 1.84176000

H 0.02393200 12.58946800 2.17492700

H -1.67741500 12.45244300 1.70336700

H -0.90884400 11.15450500 2.63416100

C -2.22722100 9.37180800 0.63397400

H -3.13676900 9.95860400 0.45042900

H -2.38793000 8.37104200 0.22602000

H -2.09643200 9.28327500 1.71541300

C 1.44923800 12.87947400 -0.29545400

H 2.43270500 12.74082400 -0.75136600

H 0.94948600 13.69024000 -0.84190900

H 1.58178200 13.21541200 0.73558500

C 1.38352800 10.87387200 -2.77001600

H 1.49223000 9.91830600 -3.28923900

H 0.87915700 11.56989800 -3.45321200

H 2.38279200 11.26989600 -2.57079100

C -0.95297700 8.77357100 -2.27791400

H -1.58380700 8.00798400 -1.82143900

H -1.55204700 9.27431300 -3.04961600

H -0.11376600 8.28593000 -2.78153900

H 4.79281100 6.08802000 -0.60969700

H 5.90765500 7.74013300 0.01709900

| **A-Gr^1+^** | |
| --- | --- |
| Energy | -2254.27637758 |

**
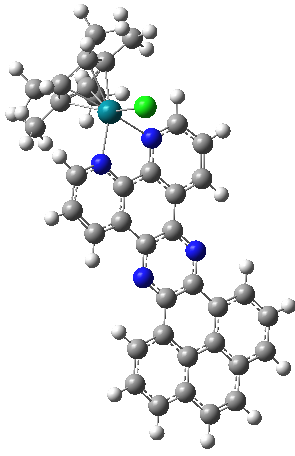
**

C -0.11514400 -0.11533500 0.66630100

C 1.23494000 -0.38920700 0.52767000

C 2.15599300 0.67291700 0.54864000

C 1.65343900 1.97948400 0.69584900

C -0.53431700 1.21406300 0.80740200

C 3.59675200 0.47718600 0.43098900

C 2.54890300 3.11278000 0.77111000

C 3.94309100 2.93430800 0.69482400

C 4.47599200 1.58977600 0.50164000

C 4.76047400 4.07189700 0.81602700

H 5.83681700 3.95966400 0.75734900

C 4.17149600 5.30830200 1.01951000

C 2.77483700 5.40080200 1.08393700

H -0.85417900 -0.90836800 0.66848900

H 1.59860100 -1.40368200 0.41300500

H -1.58229500 1.45820100 0.93009800

H 4.76894500 6.20596900 1.13040300

H 2.28664900 6.35270000 1.25391300

N 1.98427400 4.33439500 0.94780600

N 0.32518500 2.23496200 0.80575200

N 4.06890000 -0.76219600 0.25866700

N 5.79933300 1.42702100 0.39590200

C 5.38991000 -0.93166500 0.14890700

C 6.27770200 0.19166600 0.21866400

C 5.92057100 -2.27792500 -0.04591800

C 7.71944000 -0.00231500 0.09384000

C 7.32906400 -2.44794000 -0.16811300

C 8.21916600 -1.32185700 -0.09899800

C 5.07737100 -3.39144500 -0.11624700

C 5.60577600 -4.67151200 -0.30595300

H 4.93822400 -5.52646800 -0.35838300

C 6.97820400 -4.85382200 -0.42783000

H 7.38650600 -5.85008200 -0.57586300

C 7.86077300 -3.75796900 -0.36213100

C 8.60869800 1.07510900 0.15811600

C 9.98543900 0.86794700 0.03406300

H 10.66344500 1.71464200 0.08623200

C 10.48863600 -0.41370600 -0.15528200

H 11.55958100 -0.57204400 -0.25116400

C 9.62600600 -1.52505700 -0.22536700

H 4.00724200 -3.24618100 -0.02196100

H 8.21326200 2.07367200 0.30534200

Cl -0.04000200 4.05276500 3.41576900

Rh -0.17105000 4.32268600 0.96802500

C -1.43307900 4.52416700 -0.85702400

C -1.94841200 5.70146800 1.09087700

C -0.85248200 6.37180100 0.44709600

C -2.28267200 4.53383600 0.32277300

C -0.55554000 5.65336700 -0.78085000

C 10.12634400 -2.85853900 -0.42048100

H 11.19999300 -2.99733600 -0.51512900

C 9.28315100 -3.92520600 -0.48576500

H 9.67312300 -4.92878100 -0.63304700

C -0.27243200 7.68816000 0.86468700

H 0.71957300 7.85258400 0.43721000

H -0.91615500 8.50393300 0.51076400

H -0.19959700 7.77079900 1.95221800

C 0.43521300 6.07568500 -1.81900400

H 0.77171600 5.23302700 -2.42693600

H -0.03453000 6.80712700 -2.48938700

H 1.31303000 6.55360600 -1.37684300

C -1.51917200 3.54875800 -1.98782100

H -1.82870200 2.55732500 -1.64869700

H -2.26649000 3.89764600 -2.71218600

H -0.56549200 3.44993800 -2.51104300

C -3.43652300 3.61659800 0.59247500

H -4.37104100 4.09338400 0.26979300

H -3.34826300 2.67619700 0.04305300

H -3.53108600 3.38825900 1.65750200

C -2.64527400 6.16275100 2.32815200

H -1.96447100 6.67754200 3.00908600

H -3.43566900 6.86980100 2.04149600

H -3.11242600 5.33455400 2.86420200

| **A1^1-^** | |
| --- | --- |
| Energy | -1531.63629240 |

**
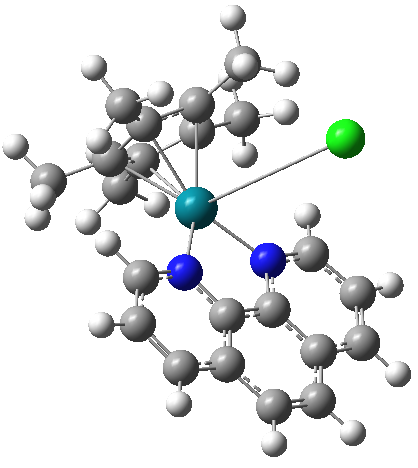
**

C 1.44210800 5.26380500 -0.65771300

C 2.76888800 4.96385400 -0.34601800

C 3.64434100 6.02165000 -0.05433500

C 3.12794300 7.34405200 -0.09310300

C 1.00743200 6.58792400 -0.67764600

C 5.03418800 5.85437400 0.28027800

C 3.95334100 8.45146200 0.18919700

C 5.32333700 8.27160400 0.51763900

C 5.83380800 6.92620500 0.55224200

C 6.09231100 9.41445400 0.78608200

H 7.14363500 9.32104100 1.04117400

C 5.47332500 10.66345600 0.71792900

C 4.12276600 10.76287300 0.38914600

H 0.73204600 4.47602700 -0.88793000

H 3.12326200 3.93759400 -0.32689800

H -0.02124300 6.82985200 -0.91837200

H 6.03068300 11.57269500 0.91935600

H 3.63572800 11.72888900 0.33419000

N 3.34332000 9.68991800 0.12145300

N 1.81482100 7.63736100 -0.40461500

Cl 1.16633200 8.39878900 4.85136100

Rh 1.35429600 9.63997600 -0.39376400

C -0.54377900 10.07522500 -1.60617500

C -0.28340700 11.05737500 0.49592200

C 0.54532500 11.75519900 -0.46019100

C -0.90960700 9.98454500 -0.20004400

C 0.30705900 11.20555700 -1.77487000

C -0.47281400 11.42869900 1.93903100

H 0.43074900 11.87945200 2.36208800

H -1.28582200 12.16049000 2.06202800

H -0.72180100 10.55846500 2.55455500

C -1.91180000 9.02091700 0.37069900

H -2.94028100 9.37172000 0.20200300

H -1.83605400 8.02938000 -0.08841300

H -1.78057000 8.89556700 1.44995000

C 1.30416900 13.02317700 -0.18124300

H 2.14972200 13.15055000 -0.86541700

H 0.65675600 13.90329600 -0.30297900

H 1.69147100 13.04910400 0.84263200

C 0.82021000 11.76443100 -3.07164300

H 0.89970100 10.99220400 -3.84362900

H 0.15282700 12.54747600 -3.46287100

H 1.81008800 12.21800600 -2.95489600

C -1.11505600 9.22058500 -2.70267900

H -1.24581400 8.18039400 -2.38566000

H -2.10479300 9.58436800 -3.01476000

H -0.47302000 9.21704000 -3.58926900

H 6.88111300 6.77997900 0.80266600

H 5.43802000 4.84602000 0.31199200

| **A2^1-^** | |
| --- | --- |
| Energy | -1455.40512993 |

**
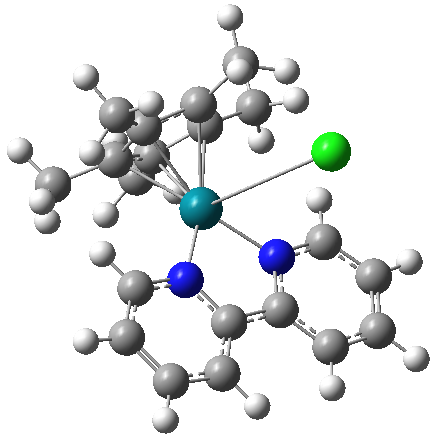
**

C 1.37399500 5.28528400 -0.65368700

C 2.69171600 4.93914700 -0.29531700

C 3.57157900 5.95406300 0.03333500

C 3.15096300 7.29728700 0.00626300

C 1.00867400 6.61546300 -0.66626100

C 3.98370200 8.43112700 0.32792600

C 5.33637400 8.35296800 0.71027300

C 6.04877400 9.50198400 0.99936200

H 7.09161700 9.44813100 1.29460700

C 5.38589300 10.74151100 0.90276000

C 4.05991800 10.76848300 0.52378700

H 0.64352300 4.52786400 -0.91856800

H 3.01022900 3.90196100 -0.27578300

H 0.00457500 6.91786100 -0.93712900

H 5.89757200 11.67327800 1.12098900

H 3.52079500 11.70326500 0.43906000

N 3.34017100 9.64895100 0.23094100

N 1.85949200 7.63005600 -0.34791500

Cl 1.14470800 8.48656900 4.90602400

Rh 1.38333000 9.61489600 -0.35354500

C -0.50211500 10.02455300 -1.59355100

C -0.25741700 11.06562000 0.47864300

C 0.57696700 11.73882500 -0.49027300

C -0.88674600 9.97825700 -0.19217700

C 0.35415100 11.14903200 -1.78711700

C -0.45848300 11.47729900 1.90956700

H 0.43711800 11.95125400 2.32408800

H -1.28044600 12.20286000 2.00634100

H -0.70064400 10.62296000 2.54955800

C -1.89725100 9.03597100 0.39902900

H -2.92336100 9.38114100 0.20600100

H -1.81704600 8.02843200 -0.02315600

H -1.77885900 8.94855200 1.48345400

C 1.32699800 13.01873200 -0.24392600

H 2.18539400 13.12483800 -0.91565900

H 0.67861200 13.89071700 -0.41043900

H 1.69401200 13.08595400 0.78545100

C 0.87658900 11.66655500 -3.09727900

H 0.97960500 10.86653400 -3.83762400

H 0.20116000 12.42173900 -3.52771000

H 1.85675200 12.14169200 -2.98477000

C -1.06764800 9.15075200 -2.67812600

H -1.28771400 8.13995400 -2.31946200

H -2.01141300 9.56157800 -3.06510500

H -0.37963900 9.05880400 -3.52484800

H 4.59196100 5.71873600 0.31491600

H 5.81799900 7.38365700 0.77730400

| **A-Gr^1-^ (two-e^-^ reduction)** | |
| --- | --- |
| Energy | -2254.52661265 |

**
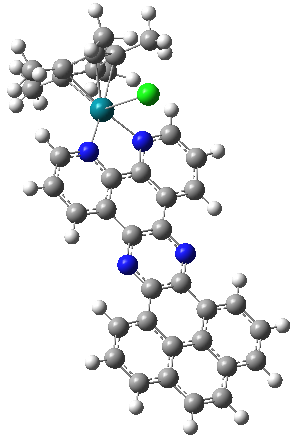
**

C -0.05118300 -0.10390000 0.27830800

C 1.31364700 -0.41535300 0.26241700

C 2.23062800 0.63503700 0.20304300

C 1.74190600 1.96185400 0.16205400

C -0.46508900 1.21984400 0.23600200

C 3.67909400 0.43999500 0.18010400

C 2.61752900 3.07697900 0.10050600

C 4.02320300 2.92061900 0.07126300

C 4.55930500 1.56093700 0.11283300

C 4.82222300 4.06333400 0.00429000

H 5.90134100 3.97009600 -0.02000100

C 4.19304100 5.31382300 -0.02941300

C 2.80813800 5.39738600 0.00450600

H -0.79968500 -0.88816000 0.32543700

H 1.66343400 -1.44017000 0.29621100

H -1.51755200 1.47412900 0.25204400

H 4.77564300 6.22780700 -0.08082200

H 2.30513800 6.35671700 -0.01724600

N 2.00035700 4.30978100 0.06855300

N 0.39641400 2.26687800 0.17819800

N 4.16364600 -0.80582400 0.22259800

N 5.88522900 1.38576200 0.08614500

C 5.48983800 -0.98163300 0.20035900

C 6.37060500 0.13925500 0.12748000

C 6.02747700 -2.33984700 0.25176100

C 7.81806600 -0.06285600 0.09580300

C 7.43942200 -2.52032400 0.22200300

C 8.32659100 -1.39215100 0.14369300

C 5.18794800 -3.45558900 0.32987300

C 5.72258200 -4.74637400 0.37819600

H 5.05646400 -5.60215300 0.43924600

C 7.09882700 -4.93905300 0.34886500

H 7.51220300 -5.94363000 0.38627300

C 7.97796400 -3.84156700 0.27095400

C 8.70528600 1.01548200 0.01942400

C 10.08628200 0.80011200 -0.01008700

H 10.76143100 1.64889800 -0.06954400

C 10.59792800 -0.49143200 0.03650100

H 11.67207700 -0.65619400 0.01396900

C 9.73807600 -1.60410800 0.11373000

H 4.11504000 -3.30196200 0.35325000

H 8.30340300 2.02172700 -0.01662400

Cl 0.25206100 2.73761500 5.39990400

Rh -0.05001800 4.26839300 0.11040800

C -1.91063200 5.08224700 -1.03276100

C -1.73959300 5.36344500 1.29361600

C -0.89289600 6.32041100 0.65810500

C -2.29386200 4.53643700 0.25221700

C -1.01276200 6.15822900 -0.78227500

C 10.24488400 -2.94864600 0.16361600

H 11.32169300 -3.09428200 0.13984000

C 9.40451800 -4.01696900 0.23920200

H 9.79992300 -5.02864100 0.27712400

C -0.16381800 7.44068800 1.34578600

H 0.71013000 7.77273000 0.77635300

H -0.81564900 8.31804000 1.46537200

H 0.17904300 7.14997100 2.34388600

C -0.39855100 7.06927700 -1.80787500

H -0.36135300 6.59926300 -2.79553900

H -0.97518100 8.00018800 -1.90853200

H 0.62428800 7.35683900 -1.54058700

C -2.42429300 4.62831800 -2.36983500

H -2.61129600 3.54961300 -2.38915700

H -3.37435500 5.12310300 -2.62178900

H -1.71742900 4.85471700 -3.17444000

C -3.32318000 3.46080800 0.46302400

H -4.33182000 3.89226100 0.53197500

H -3.33883600 2.74336100 -0.36389400

H -3.14642100 2.90484600 1.38976600

C -2.03857900 5.26284000 2.76145400

H -2.23242200 4.22974300 3.06619400

H -1.20909000 5.63313600 3.37208700

H -2.92886400 5.85255500 3.02880100

| **A-Gr^0^ (one-e^-^ reduction)** | |
| --- | --- |
| Energy | -2254.40435420 |


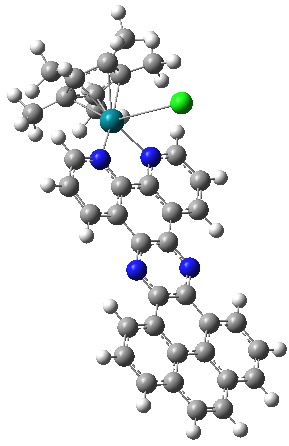


C -0.06484500 -0.13436400 0.25655700

C 1.29150400 -0.41842500 0.23903800

C 2.21196800 0.64354700 0.26774900

C 1.70787200 1.95704500 0.31174500

C -0.48659400 1.19893700 0.29765300

C 3.65681200 0.43924500 0.25082000

C 2.60284100 3.09159300 0.34931800

C 3.99867600 2.90985200 0.34500500

C 4.53605700 1.55433900 0.28962100

C 4.81569200 4.05271200 0.39180000

H 5.89302600 3.93564400 0.38976400

C 4.22164000 5.30392900 0.44251800

C 2.82611200 5.40125500 0.44077300

H -0.80557900 -0.92586400 0.23905100

H 1.65769700 -1.43783200 0.20519800

H -1.53979500 1.44966100 0.31385200

H 4.81800500 6.20836200 0.48423700

H 2.33378600 6.36476700 0.48443000

N 2.02841400 4.32714400 0.39141600

N 0.37139600 2.22641300 0.32185300

N 4.13372500 -0.80906200 0.19396600

N 5.86255500 1.38341300 0.27250600

C 5.45851200 -0.98602100 0.17434900

C 6.34493800 0.13811400 0.21587700

C 5.99423800 -2.34312000 0.10631100

C 7.79128500 -0.06445100 0.19392400

C 7.40665200 -2.52201700 0.08194100

C 8.29620400 -1.39410100 0.12573200

C 5.15211500 -3.45873700 0.06310700

C 5.68524400 -4.74916300 -0.00439900

H 5.01808600 -5.60546600 -0.03729200

C 7.06164400 -4.94030200 -0.02938400

H 7.47369900 -5.94465800 -0.08173900

C 7.94333700 -3.84266500 0.01298300

C 8.67997800 1.01447400 0.23673700

C 10.06087200 0.79881800 0.21237100

H 10.73811600 1.64712600 0.24648400

C 10.56923000 -0.49296200 0.14480900

H 11.64333800 -0.65781400 0.12557000

C 9.70734500 -1.60614000 0.10069700

H 4.07897900 -3.30655400 0.08250600

H 8.28056300 2.02091100 0.28928100

Cl 0.74089400 3.10991800 4.67178800

Rh -0.09526600 4.30083900 0.35284100

C -1.85306300 4.91356700 -0.96725300

C -1.79527500 5.53170400 1.29144300

C -0.89537700 6.38120300 0.56402400

C -2.31801600 4.56882500 0.36263800

C -0.96854300 6.02813900 -0.84117500

C 10.21248500 -2.95024200 0.03123800

H 11.28933500 -3.09551800 0.01349300

C 9.36987600 -4.01853300 -0.01091100

H 9.76325400 -5.03021900 -0.06306000

C -0.17940200 7.57119400 1.13368900

H 0.65157400 7.89239600 0.49916000

H -0.86646400 8.42399300 1.21626500

H 0.21345500 7.36742800 2.13440300

C -0.29143100 6.75614900 -1.96357000

H -0.13437500 6.10769700 -2.82995500

H -0.90481300 7.60625400 -2.29237800

H 0.68090500 7.15654600 -1.66220900

C -2.27323200 4.24913700 -2.24361700

H -2.45944600 3.18051500 -2.10226600

H -3.20413000 4.69530100 -2.62024000

H -1.51575000 4.35849700 -3.02486000

C -3.35644700 3.53273300 0.68172200

H -4.35658500 3.98640900 0.68416000

H -3.37276700 2.72875200 -0.05976300

H -3.19678500 3.08667600 1.66793700

C -2.12696700 5.63259700 2.74824900

H -1.28792700 6.02662800 3.32853300

H -2.98017300 6.30938800 2.89974400

H -2.39960400 4.66103600 3.16939700

C 1.44210800 5.26380500 -0.65771300

| **TS: A1…H^+^…N(Eth)_3_** | |
| --- | --- |
| Energy | -1364.11220441 |

**
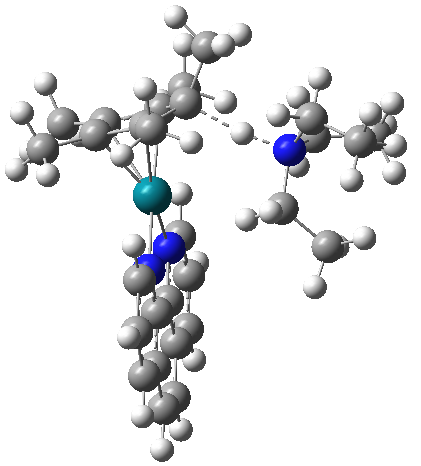
**

C -0.38356300 0.93632800 -0.77602600

N -1.04636700 1.89215000 -0.05023000

C -1.97678600 1.47816000 0.82019500

C -2.28414200 0.12464300 1.02091400

C -1.61140100 -0.84789600 0.29942900

C 2.43289500 2.36746700 -3.48844700

C 2.26599900 0.99482300 -3.40442100

C 0.59123200 1.39970700 -1.70786700

N 0.76952100 2.75654000 -1.78967400

C 1.66956400 3.21298800 -2.67010000

Rh -0.42122300 3.85147500 -0.44825500

C 0.57348700 5.67940500 0.35303500

C -0.37842800 5.19779300 1.32473800

C -1.68062500 5.32930200 0.74739600

C -1.61963100 6.08223300 -0.52834200

C -0.16579300 6.11245200 -0.79183500

C 2.05911100 5.79156700 0.55458600

C -0.05456000 4.72492000 2.71463800

C -2.95741500 5.07065600 1.50016800

C 0.44897700 6.80852800 -1.97489200

C -2.38441500 7.41796200 -0.58471900

H -3.46457300 7.28393100 -0.46166400

H -2.04598500 8.08624000 0.22322200

H -2.41527900 5.39722700 -1.74577700

H -2.22246500 7.94416800 -1.53002500

C 1.32044300 0.47579300 -2.49543500

C -0.63003600 -0.45086700 -0.63253000

H 2.60718000 5.66594500 -0.38395700

H 2.32326400 6.78082500 0.95387700

H 2.43125200 5.04198600 1.25949300

H -0.75241900 3.95432000 3.05546300

H 0.95526500 4.30812800 2.77616700

H -0.11342900 5.55589700 3.43159000

H -3.73280100 4.63418100 0.86393100

H -2.80656200 4.40370000 2.35449800

H -3.35963900 6.01291500 1.89928600

H -0.09009900 6.59700000 -2.90393900

H 0.42721500 7.89794000 -1.82836200

H 1.49723000 6.53136100 -2.12338900

H -2.49123100 2.25281500 1.37606300

H -3.04813000 -0.14047300 1.74367300

H -1.83033800 -1.90202700 0.44015400

H 3.14640600 2.80525500 -4.17810000

H 2.84717000 0.32057300 -4.02620100

H 1.78615300 4.28902100 -2.72299000

N -3.14675300 5.02641600 -2.69819700

C -4.39373400 4.58811600 -1.98562600

C -5.68319100 4.46004700 -2.79657700

H -4.55348000 5.32148900 -1.19015400

H -4.14647800 3.63967700 -1.50028000

H -6.47185800 4.11914800 -2.11685700

H -6.00242000 5.42178300 -3.20794000

H -5.60916300 3.73724200 -3.61134400

C -3.30555300 6.24321500 -3.56372700

C -3.73501200 6.03258100 -5.01636200

H -2.33235400 6.74299100 -3.55603300

H -4.00877400 6.90255800 -3.04867000

H -3.80285800 7.01765100 -5.49091000

H -3.00020600 5.44992800 -5.57905700

H -4.70979400 5.54950900 -5.10961400

C -2.35340800 3.92201500 -3.31858600

C -3.09410500 2.84714600 -4.11277900

H -1.82026600 3.45057700 -2.47996600

H -1.59038100 4.39989600 -3.93979700

H -2.34838500 2.13469300 -4.48269000

H -3.79693100 2.28596200 -3.49004400

H -3.63208300 3.24376700 -4.97712600

C 1.05826300 -0.92741900 -2.32925300

H 1.62172200 -1.63254200 -2.93320400

C 0.12481000 -1.37063700 -1.43829700

H -0.06566600 -2.43355700 -1.32280700

| **TS: A2…H^+^…N(Eth)_3_** | |
| --- | --- |
| Energy | -1287.88027330 |

**
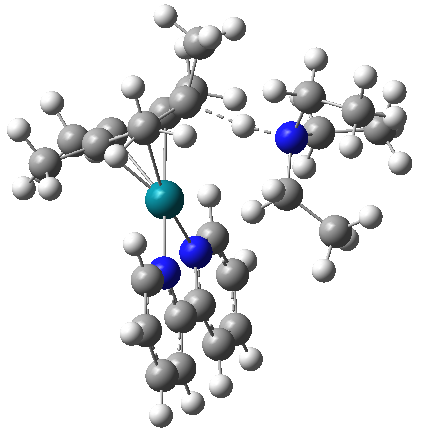
**

C -0.50865800 0.95017100 -0.78795800

N -1.13083300 1.95200700 -0.09684100

C -2.12483200 1.62601200 0.75771300

C -2.53739700 0.31858900 0.97220300

C -1.89930000 -0.71257400 0.27644600

C 2.47058100 2.43749000 -3.38447300

C 2.29449000 1.05141200 -3.33403800

C 0.54579800 1.39578900 -1.70462800

N 0.73291900 2.74893500 -1.74932200

C 1.67460200 3.24233800 -2.58184700

Rh -0.44127800 3.87973900 -0.45053400

C 0.59518000 5.70182600 0.31753900

C -0.34842500 5.24549400 1.31180700

C -1.65659000 5.39675700 0.75852700

C -1.60387300 6.13973300 -0.52454300

C -0.15627100 6.15288800 -0.81045500

C 2.08715200 5.78346900 0.48777100

C -0.00686700 4.77901500 2.69970500

C -2.92317000 5.17893700 1.54101900

C 0.44356900 6.83488400 -2.00899600

C -2.36292000 7.47907000 -0.57364800

H -3.44457300 7.34570200 -0.46304900

H -2.03025100 8.13644600 0.24524800

H -2.39564700 5.47202100 -1.74056400

H -2.19003200 8.01545200 -1.51108600

C 1.32223000 0.53215800 -2.48761200

C -0.87931900 -0.38880900 -0.60994100

H 2.61429600 5.62288800 -0.45788000

H 2.38310800 6.77450500 0.85902700

H 2.45487100 5.04078200 1.20213200

H -0.71796800 4.03168700 3.06412300

H 0.99245300 4.33553100 2.74466600

H -0.02728000 5.61933000 3.40796400

H -3.72989500 4.77274700 0.92383000

H -2.77459500 4.50316400 2.38885800

H -3.28232700 6.13264400 1.95374500

H -0.10981200 6.61486400 -2.92762800

H 0.42554500 7.92559000 -1.87291500

H 1.48910700 6.55519800 -2.17002400

H -2.58905900 2.45448700 1.27772800

H -3.34131100 0.11788200 1.67194600

H -2.19266500 -1.74718000 0.42050200

H 3.21209700 2.89197200 -4.03239600

H 2.90151500 0.38963100 -3.94293900

H 1.77876900 4.32032400 -2.58911500

H 1.16880300 -0.53894800 -2.43374000

H -0.37502400 -1.17211200 -1.16294800

N -3.12382400 5.08934800 -2.69978200

C -4.27380300 4.46583200 -1.96451000

C -5.57535200 4.21453300 -2.72582200

H -4.48538400 5.13926900 -1.12867100

H -3.88845300 3.53693600 -1.53430900

H -6.27836900 3.73654700 -2.03471300

H -6.03594700 5.14727800 -3.06336200

H -5.45115200 3.55355200 -3.58544100

C -3.46691600 6.32907400 -3.47410900

C -3.95347300 6.15560400 -4.91367000

H -2.55715200 6.93639600 -3.48497200

H -4.20892800 6.87097300 -2.88185100

H -4.16496400 7.15187700 -5.31734200

H -3.18912100 5.70207300 -5.55111000

H -4.86808500 5.56396100 -4.98998600

C -2.23920800 4.13049300 -3.43024100

C -2.88644700 3.02085100 -4.25798100

H -1.61003600 3.67618600 -2.65149400

H -1.57747200 4.73641700 -4.05647100

H -2.08079600 2.43036600 -4.70839300

H -3.47968500 2.34070300 -3.64002500

H -3.51584500 3.39643800 -5.06840500

| **TS: A-Gr…H^+^…N(Eth)_3_ (two-e^-^ reduction)** | |
| --- | --- |
| Energy | -2087.00111755 |


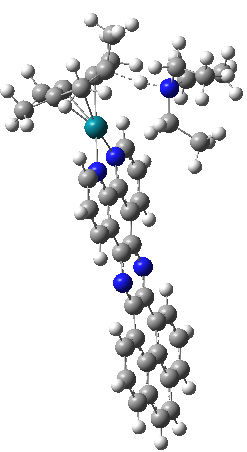


C -0.29118200 0.99757800 -0.69198800

N -0.97795600 1.95893700 -0.00404200

C -1.92226800 1.55624600 0.86301200

C -2.21508700 0.20946000 1.09110900

C -1.51234400 -0.77244600 0.40476800

C 2.54774100 2.46708000 -3.37995700

C 2.42097100 1.08808000 -3.27278900

C 0.69901500 1.46750000 -1.62433200

N 0.83494900 2.82414300 -1.72743000

C 1.74215700 3.29822400 -2.59749600

Rh -0.39701400 3.92138200 -0.43205400

C 0.55546700 5.79447000 0.31021400

C -0.37051600 5.31336900 1.30743200

C -1.68264400 5.39462200 0.74475800

C -1.65838700 6.11829300 -0.54968000

C -0.21027100 6.18292800 -0.83314100

C 2.04098000 5.94630200 0.48554800

C -0.01426500 4.88394900 2.70340000

C -2.94214900 5.12219000 1.52097300

C 0.36763800 6.86190400 -2.04426700

C -2.46373500 7.42937500 -0.62476300

H -3.53801800 7.26452400 -0.48835300

H -2.13805700 8.12224300 0.16731900

H -2.43854400 5.39092300 -1.74347200

H -2.32605000 7.94178900 -1.58127400

C 1.47483200 0.56438700 -2.37814400

C -0.52487900 -0.38020200 -0.51205200

H 2.57787700 5.80578900 -0.45758600

H 2.28842400 6.95219600 0.85234400

H 2.44034800 5.22548800 1.20529400

H -0.70092600 4.12127600 3.08243900

H 0.99888400 4.47402600 2.75582800

H -0.06216000 5.73647100 3.39541600

H -3.72008700 4.66563700 0.90215900

H -2.76588100 4.46745400 2.37994900

H -3.35401600 6.06169000 1.91662200

H -0.17277400 6.60053800 -2.95974400

H 0.30783900 7.95391800 -1.93262400

H 1.42355700 6.61781200 -2.19577100

H -2.45323400 2.34108700 1.38755200

H -2.98851600 -0.05116100 1.80530600

H -1.70950100 -1.82658600 0.56017700

H 3.26315000 2.91244500 -4.06265300

H 3.03018900 0.41352600 -3.86268800

H 1.82228700 4.37665500 -2.66356300

N -3.16344300 4.97584800 -2.68695400

C -4.37470900 4.47948700 -1.95170900

C -5.66590800 4.26728900 -2.74188800

H -4.56482400 5.21645200 -1.16613200

H -4.06874500 3.55458500 -1.45430700

H -6.42454200 3.89256600 -2.04597200

H -6.04451000 5.20366300 -3.16121600

H -5.56300600 3.53807900 -3.54771000

C -3.39933700 6.17392600 -3.56080600

C -3.83701900 5.92571500 -5.00482400

H -2.45448800 6.72537300 -3.57200200

H -4.12977000 6.79988000 -3.04203000

H -3.96368600 6.90071000 -5.48800700

H -3.08029000 5.37674300 -5.57231200

H -4.78616100 5.39094400 -5.07897200

C -2.31823600 3.91193300 -3.30852800

C -3.00716600 2.78862400 -4.08237900

H -1.74841100 3.47957600 -2.47277800

H -1.59275000 4.42603100 -3.94583800

H -2.22695400 2.11738500 -4.45812500

H -3.66660000 2.19342700 -3.44399400

H -3.58064400 3.14563900 -4.94120200

C 1.25750800 -0.86794100 -2.19543500

C 0.26980100 -1.33177000 -1.28296000

N 1.99942600 -1.73095700 -2.89862400

C 1.79162200 -3.04075700 -2.72776900

C 0.79112300 -3.50689800 -1.81703000

C 2.60091800 -3.99456000 -3.48256200

N 0.05388200 -2.64092800 -1.11409500

C 0.56379800 -4.93938500 -1.64305400

C 2.37052400 -5.38746600 -3.29642900

C 3.58970000 -3.56789800 -4.37478000

C 1.36077600 -5.85527900 -2.38685500

C -0.41293900 -5.42215700 -0.76638000

C 3.15533600 -6.33022300 -4.02602200

C 4.35451200 -4.49730500 -5.08566600

H 3.75387400 -2.50427900 -4.50543600

C 1.15120300 -7.25768200 -2.22382500

C -0.61366700 -6.79701800 -0.61338700

H -1.01203200 -4.71245200 -0.20724500

C 4.14166600 -5.86014500 -4.91486700

C 2.91814000 -7.73523700 -3.83518800

H 5.11888900 -4.14999300 -5.77459600

C 0.15729600 -7.70385200 -1.33103100

C 1.96057800 -8.17820700 -2.97493100

H -1.37658200 -7.15611800 0.07109500

H 4.73763300 -6.58068000 -5.46891000

H 3.52408100 -8.44085400 -4.39731400

H -0.00028800 -8.77234900 -1.20954000

H 1.79028100 -9.24300000 -2.83979300

| **TS: A-Gr…H^+^…N(Eth)_3_ (one-e^-^ reduction)** | |
| --- | --- |
| Energy | -2086.84034394 |

**
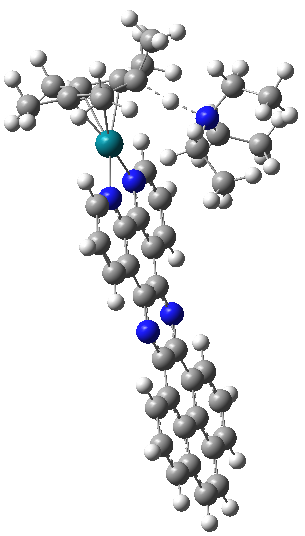
**

C -0.24446000 1.16337400 -0.45232600

N -0.90047000 2.11181100 0.27159100

C -1.84639400 1.72916200 1.13425400

C -2.19680900 0.38485300 1.31330700

C -1.54790500 -0.58799400 0.57287300

C 2.78214500 2.56399000 -2.99241800

C 2.53805800 1.20168300 -2.96557400

C 0.79349000 1.61857100 -1.35298600

N 1.03120800 2.95843100 -1.40198600

C 2.00435200 3.41444100 -2.19654500

Rh -0.22503900 4.10740500 -0.09992600

C 0.45194000 6.16259500 0.49130700

C -0.55950900 5.71441600 1.43476000

C -1.78461600 5.58694500 0.73462400

C -1.64059500 6.09519900 -0.65959700

C -0.16998300 6.31236100 -0.77338200

C 1.87274600 6.48107700 0.84774400

C -0.32179700 5.49395600 2.89781800

C -3.10551400 5.29673600 1.37952100

C 0.48798100 6.89559100 -1.98644200

C -2.52415800 7.32150500 -0.99840200

H -3.58812300 7.08200900 -0.94046300

H -2.31065000 8.11619600 -0.27235100

H -2.17676100 5.24450100 -1.67739900

H -2.30517300 7.70788900 -1.99567300

C 1.52334200 0.70015200 -2.12954700

C -0.54348200 -0.20622800 -0.33577200

H 2.52999500 6.45159400 -0.02424600

H 1.92764600 7.49259500 1.27097600

H 2.26647200 5.78906800 1.59764300

H -1.08044100 4.84659100 3.34269200

H 0.66070700 5.05049900 3.08380500

H -0.35452400 6.45659600 3.42484100

H -3.80837900 4.81810000 0.69435900

H -3.00598500 4.67222800 2.27087300

H -3.55899500 6.24390500 1.70298900

H 0.06895800 6.50310700 -2.91543900

H 0.32993300 7.98280400 -1.98865500

H 1.56860700 6.73269100 -1.99363000

H -2.34091500 2.51332200 1.69345500

H -2.97204000 0.12778000 2.02579800

H -1.79398200 -1.63824000 0.67786400

H 3.55843400 2.98577300 -3.62025400

H 3.11181600 0.51108300 -3.57258100

H 2.16194500 4.48565900 -2.20590500

N -2.80435100 4.55590100 -2.74885900

C -3.38715900 3.37753800 -2.03660900

C -4.47652000 2.55749700 -2.73365500

H -3.80003100 3.76280300 -1.09986800

H -2.54582100 2.73494900 -1.76953600

H -4.74667200 1.73236100 -2.06522400

H -5.38221000 3.14538500 -2.90594100

H -4.15137300 2.12560900 -3.68241000

C -3.81169100 5.48345500 -3.35579500

C -4.33069400 5.15889600 -4.76103800

H -3.34229400 6.46775000 -3.39401200

H -4.64301600 5.54736200 -2.64756600

H -5.06511000 5.92670100 -5.02826800

H -3.53171000 5.20005900 -5.50686800

H -4.82280600 4.18720400 -4.82894900

C -1.64033400 4.25008600 -3.63223500

C -1.73133300 3.04055000 -4.56600700

H -0.78517200 4.09937500 -2.96449000

H -1.44371200 5.15862600 -4.20936300

H -0.79657700 2.98633500 -5.13463000

H -1.82999800 2.10368600 -4.01035500

H -2.55391700 3.11294700 -5.28054200

C 1.20172800 -0.71984900 -2.04134100

C 0.18764600 -1.16615400 -1.15481500

N 1.87172000 -1.58454200 -2.81118500

C 1.56403700 -2.88217000 -2.72812700

C 0.54622700 -3.33442700 -1.82378100

C 2.27253500 -3.83796200 -3.57413200

N -0.11822600 -2.46455700 -1.05773600

C 0.22091900 -4.75457600 -1.73374000

C 1.93629800 -5.21853900 -3.47818800

C 3.26210300 -3.42421200 -4.47171100

C 0.92270300 -5.67232000 -2.56611200

C -0.76077700 -5.22334000 -0.85487600

C 2.61780900 -6.16292300 -4.30267000

C 3.92429400 -4.35522400 -5.27692900

H 3.50667100 -2.36995000 -4.53440000

C 0.61042100 -7.06274400 -2.49190600

C -1.05936100 -6.58702700 -0.78620800

H -1.28675500 -4.51264200 -0.22767500

C 3.60775100 -5.70601200 -5.19465100

C 2.27655400 -7.55571800 -4.20232000

H 4.68956000 -4.01872600 -5.97003400

C -0.38450800 -7.49519800 -1.59324800

C 1.31800300 -7.98574200 -3.33669000

H -1.82361900 -6.93620500 -0.09830300

H 4.12410900 -6.42760400 -5.82221600

H 2.80443800 -8.26242200 -4.83695400

H -0.61982800 -8.55479900 -1.53861300

H 1.06867300 -9.04127300 -3.26936900

| **A1-[η^4^-Cp*H]** | |
| --- | --- |
| Energy | -1532.12342476 |

**
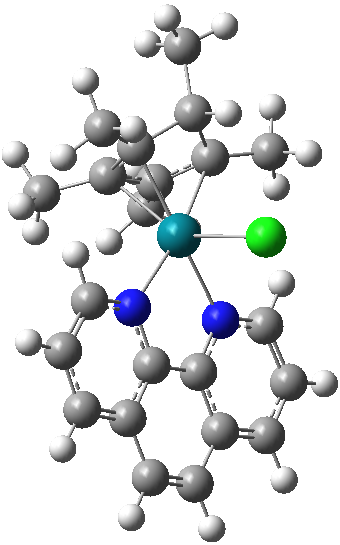
**

Rh -1.03530400 0.19174400 0.08535600

Cl 1.44200600 0.20010700 1.17769200

N -0.24079700 1.54490800 -1.44637900

C -2.00176200 0.19708100 2.59475100

H -1.00137800 0.19794500 3.04365800

C -2.13518200 1.33652000 1.58522900

C -3.02320000 0.91560800 0.53347000

C -3.04850100 0.19933100 3.72991800

H -2.93369500 1.08473700 4.36629800

H -2.93375200 -0.68357400 4.36975600

C -1.89651600 2.76405100 1.99265600

H -0.89377300 2.89247600 2.41561000

H -2.61479600 3.07078100 2.76747100

H -2.00836200 3.46666300 1.16095800

C -3.84404700 1.79675100 -0.36187300

H -4.05587600 1.32012400 -1.32334400

H -3.34271000 2.74859500 -0.55981100

H -4.80587400 2.02833700 0.11605900

C -0.22493200 2.87658300 -1.43112000

H -0.67273100 3.35321900 -0.56703500

C 0.33716900 3.65046000 -2.46103300

H 0.31232500 4.73224200 -2.38808500

C 0.91100800 3.01470400 -3.54462300

H 1.35446200 3.58230800 -4.35750400

C 0.92363600 1.60346400 -3.58907200

C 0.33029000 0.90407100 -2.50689900

N -0.24166300 -1.16885400 -1.44009500

C -0.22760800 -2.50052300 -1.41940500

H -0.67565900 -2.97301900 -0.55319600

C 0.33281800 -3.27939000 -2.44645600

H 0.30641900 -4.36082700 -2.36908500

C 0.90711400 -2.64890500 -3.53287500

H 1.34949400 -3.22045800 -4.34356600

C 0.92166500 -1.23788900 -3.58314000

C 0.32952900 -0.53320500 -2.50374700

C -2.13522500 -0.94617600 1.58955300

C -3.02336400 -0.52914800 0.53615800

H -4.06757300 0.19855500 3.32438400

C -1.89707900 -2.37220500 2.00260900

H -0.89375300 -2.49974400 2.42447400

H -2.61437400 -2.67512500 2.77983300

H -2.01087100 -3.07819000 1.17404700

C -3.84507900 -1.41334300 -0.35534800

H -4.05609200 -0.94084000 -1.31903800

H -3.34493000 -2.36671900 -0.54883400

H -4.80732000 -1.64151000 0.12340000

C 1.50862900 0.86155500 -4.67004600

H 1.95638800 1.41293900 -5.49157000

C 1.50759900 -0.50129100 -4.66723600

H 1.95442700 -1.05673400 -5.48653300

| **A2-[η^4^-Cp*H]** | |
| --- | --- |
| Energy | -1455.89045422 |

**
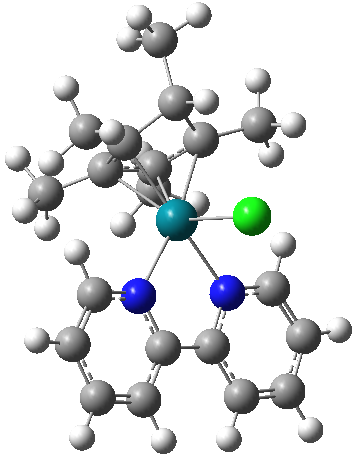
**

Rh -1.02644800 0.18987600 0.05207600

Cl 1.45388200 0.17244300 1.15630400

N -0.23590800 1.52471500 -1.47605300

C -1.98134100 0.18676800 2.57050100

H -0.97754600 0.17736400 3.01149800

C -2.11611900 1.33449700 1.57131100

C -3.00852600 0.92617700 0.52050200

C -3.01892600 0.18983900 3.71441000

H -2.89239500 1.07045700 4.35510700

H -2.90536800 -0.69751900 4.34819600

C -1.87041200 2.75675800 1.99332100

H -0.85734000 2.88230800 2.39200100

H -2.56887500 3.04832900 2.79161100

H -2.00742400 3.47279100 1.17712600

C -3.82863400 1.81792800 -0.36519300

H -4.04749600 1.34916300 -1.32893300

H -3.32371400 2.76871000 -0.55918400

H -4.78698400 2.05130800 0.11879800

C -0.25378800 2.86619100 -1.41465300

H -0.70177000 3.29418600 -0.52655400

C 0.26731300 3.68077500 -2.41480600

H 0.22160800 4.75937100 -2.31182100

C 0.83809300 3.07519000 -3.53330600

H 1.25306100 3.67111900 -4.33976800

C 0.87229300 1.68605100 -3.60019900

H 1.31537900 1.20290700 -4.46197000

C 0.33227100 0.92554500 -2.55468700

N -0.23428500 -1.14853500 -1.47014900

C -0.23270500 -2.48923400 -1.39241500

H -0.69583900 -2.91355100 -0.51038900

C 0.32782700 -3.30732300 -2.36807900

H 0.29618200 -4.38519700 -2.25296000

C 0.92370000 -2.70600300 -3.47571700

H 1.37584700 -3.30465500 -4.25987600

C 0.93599700 -1.31742100 -3.55999500

H 1.40500400 -0.83755200 -4.40977100

C 0.34830500 -0.55332000 -2.54325400

C -2.13284900 -0.95015200 1.56137700

C -3.01896800 -0.51941400 0.51363600

H -4.04122000 0.19888600 3.31715500

C -1.91018300 -2.37959700 1.97168400

H -0.89964300 -2.52497400 2.37019400

H -2.61384500 -2.66658400 2.76708700

H -2.05824800 -3.08646700 1.14939700

C -3.85263900 -1.39028500 -0.38019100

H -4.06151200 -0.91018900 -1.34057300

H -3.36328600 -2.34778800 -0.58082000

H -4.81590000 -1.61099600 0.10002300

| **A-[η^4^-Cp*H]-Gr (two-e^-^ reduction)** | |
| --- | --- |
| Energy | -2255.01207879 |

**
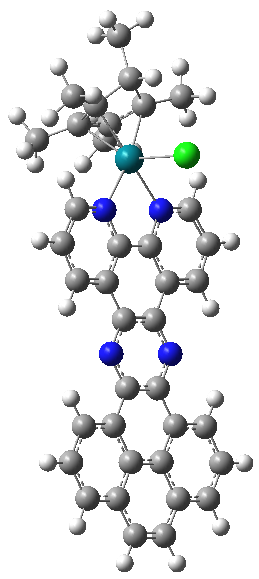
**

Rh -3.50228100 1.12866500 5.45329800

Cl -1.13814800 1.19047600 6.75183100

N -2.62966200 2.48567300 3.97284400

C -4.64599000 1.12001900 7.88566700

H -3.67753700 1.13475400 8.39952300

C -4.72583100 2.25774900 6.86873200

C -5.52834900 1.82451100 5.75575200

C -5.76716300 1.10722800 8.94695900

H -5.70830300 1.99359600 9.58966400

H -5.68370700 0.22561300 9.59346000

C -4.53709500 3.68839200 7.29102300

H -3.56716600 3.83159600 7.78028700

H -5.31051800 3.98245500 8.01597600

H -4.60458500 4.39056900 6.45417500

C -6.29433800 2.69371400 4.80199300

H -6.42819500 2.21461200 3.82782200

H -5.79482000 3.65337400 4.64079300

H -7.29205300 2.91011900 5.20801900

C -2.63940400 3.82115800 4.00112400

H -3.13634400 4.27894700 4.84787200

C -2.04523800 4.60995400 3.00855300

H -2.09022600 5.69073200 3.08556900

C -1.41031000 3.98965300 1.94611000

H -0.93830300 4.55934000 1.15420000

C -1.37415100 2.58562500 1.89900700

C -1.99781400 1.86557100 2.94002500

N -2.58380700 -0.21408200 3.99016500

C -2.54860100 -1.54896700 4.03439600

H -3.02797200 -2.01312200 4.88777700

C -1.93203400 -2.32928000 3.04883600

H -1.94131700 -3.40997600 3.13857700

C -1.32154000 -1.70108500 1.97673100

H -0.83378500 -2.26424000 1.18969500

C -1.33079700 -0.29726900 1.91369400

C -1.97453900 0.41419600 2.94842600

C -4.69431500 -0.02484900 6.87480400

C -5.50834600 0.38049500 5.75909000

H -6.75637800 1.09237400 8.47347400

C -4.47122500 -1.44781700 7.30612400

H -3.50411600 -1.56219900 7.80828500

H -5.24514800 -1.76018000 8.02294200

H -4.50903900 -2.15530800 6.47186900

C -6.25169700 -0.51419600 4.81107300

H -6.39371500 -0.04663800 3.83244800

H -5.72972100 -1.46344300 4.65955400

H -7.24540200 -0.75010300 5.21616600

C -0.70091600 0.43841800 0.82241800

C -0.71910800 1.85828800 0.81712100

N -0.11119900 -0.24376000 -0.16596500

N -0.14163900 2.54809500 -0.17314600

C 0.46282800 0.44045700 -1.16078600

C 0.45015200 1.87153200 -1.16271000

C 1.10311500 -0.29116500 -2.25139900

C 1.08386600 2.61170100 -2.25134200

C 1.71096500 0.44577800 -3.30737800

C 1.70246500 1.88301300 -3.30675100

C 1.12722500 -1.68915500 -2.27771700

C 1.74232000 -2.36990000 -3.33240200

H 1.75202800 -3.45589800 -3.33823400

C 2.33950200 -1.66486300 -4.37062400

H 2.81703600 -2.19640200 -5.18962600

C 2.33736900 -0.25643300 -4.38056800

C 1.09249800 4.00991100 -2.27563400

C 1.70267200 4.69891400 -3.32780400

H 1.70128100 5.78496300 -3.33159100

C 2.30909000 4.00198100 -4.36615500

H 2.78237100 4.53990300 -5.18346300

C 2.32203500 2.59362400 -4.37836400

H 0.66039300 -2.23683300 -1.46698500

H 0.61832400 4.55120400 -1.46486200

C 2.94340300 1.85336900 -5.44254100

H 3.41079800 2.40851200 -6.25150500

C 2.95037000 0.49213200 -5.44380700

H 3.42335000 -0.05666400 -6.25385900

| **A-[η^4^-Cp*H]-Gr (one-e^-^ reduction)** | |
| --- | --- |
| Energy | -2254.84651618 |

**
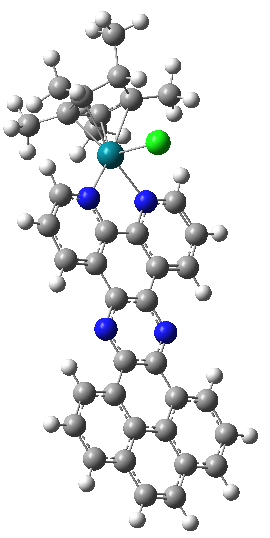
**

Rh -3.77622900 1.12296200 5.10567900

Cl -1.49259900 1.15594500 6.34024400

N -2.86533100 2.47556000 3.72786600

C -4.38963400 1.12989200 7.84231200

H -3.32832700 1.14763300 8.09656200

C -4.76861900 2.28316000 6.92320800

C -5.72217500 1.83312800 5.99221200

C -5.21697100 1.12508000 9.15126000

H -4.99036500 2.01661200 9.74447400

H -4.96230000 0.24904900 9.75599900

C -4.44989000 3.70283800 7.26730100

H -3.37649700 3.82614000 7.44396100

H -4.96522700 3.98220700 8.19627900

H -4.76808200 4.40766800 6.49518500

C -6.67594100 2.65809100 5.18021000

H -6.82019400 2.25215300 4.17509700

H -6.34584800 3.69524500 5.09204600

H -7.65526500 2.66267700 5.67690700

C -2.87740700 3.80880400 3.77973400

H -3.40437700 4.25995400 4.61079800

C -2.23656300 4.60067100 2.81793800

H -2.27936800 5.68033300 2.90382900

C -1.55899400 3.98682400 1.77892900

H -1.04927000 4.56334500 1.01582300

C -1.52393500 2.58193900 1.71580200

C -2.19470700 1.85827400 2.71904400

N -2.82960200 -0.21685000 3.73699900

C -2.80910500 -1.54960100 3.79657800

H -3.32407100 -2.00836200 4.63109800

C -2.15136100 -2.33112800 2.83770700

H -2.16804100 -3.41103500 2.92943600

C -1.49103100 -1.70682500 1.79378900

H -0.96919100 -2.27514100 1.03271300

C -1.48992700 -0.30185000 1.72285600

C -2.17652200 0.41086100 2.72328400

C -4.73265800 -0.04580200 6.93765900

C -5.69906300 0.36301000 6.00098100

H -6.29220900 1.10673200 8.94358800

C -4.36938500 -1.45041300 7.29902900

H -3.29283400 -1.53747000 7.47780600

H -4.87655100 -1.73446600 8.23107300

H -4.66414900 -2.17444700 6.53547200

C -6.62413400 -0.50020500 5.19569900

H -6.75566400 -0.12462800 4.17695600

H -6.27460500 -1.53327500 5.14185600

H -7.61240500 -0.50783700 5.67405100

C -0.80161000 0.43596700 0.66942300

C -0.81679700 1.85530500 0.66704400

N -0.15464700 -0.24598500 -0.28215200

N -0.18194300 2.54774800 -0.28506000

C 0.48172100 0.43935600 -1.23669100

C 0.46918200 1.87298300 -1.23717100

C 1.19418100 -0.29001600 -2.28195700

C 1.17205500 2.61408400 -2.28062900

C 1.87258000 0.44920200 -3.29242400

C 1.86219100 1.88622200 -3.29139500

C 1.22243600 -1.68800500 -2.30645700

C 1.91108100 -2.36682100 -3.31582000

H 1.92382300 -3.45269800 -3.32134600

C 2.57786900 -1.65966400 -4.30924200

H 3.11285900 -2.18986400 -5.09276900

C 2.57371700 -0.25111100 -4.31931800

C 1.18060400 4.01237500 -2.30274500

C 1.86110000 4.70254900 -3.30991500

H 1.85911400 5.78850600 -3.31323300

C 2.53860100 4.00654800 -4.30397200

H 3.06699700 4.54552500 -5.08599100

C 2.55394700 2.59809800 -4.31666000

H 0.70216000 -2.23711800 -1.52997200

H 0.65186200 4.55280800 -1.52588700

C 3.24906300 1.85982100 -5.33553800

H 3.77055000 2.41643400 -6.10956000

C 3.25829600 0.49863600 -5.33696000

H 3.78720100 -0.04920400 -6.11221000

1 Frisch, M. J.; Trucks, G. W.; Schlegel, H. B.; Scuseria, G. E.; Robb, M. A.; Cheeseman, J.

R.; Scalmani, G.; Barone, V.; Mennucci, B.; Petersson, G. A.; Nakatsuji, H.; Caricato, M.;

Li, X.; Hratchian, H. P.; Izmaylov, A. F.; Bloino, J.; Zheng, G.; Sonnenberg, J. L.; Hada,

M.; Ehara, M.; Toyota, K.; Fukuda, R.; Hasegawa, J.; Ishida, M.; Nakajima, T.; Honda,

Y.; Kitao, O.; Nakai, H.; Vreven, T.; Montgomery, J. A. J.; Peralta, J. E.; Ogliaro, F.;

Bearpark, M.; Heyd, J. J.; Brothers, E.; Kudin, K. N.; Staroverov, V. N.; Kobayashi, R.;

Normand, J.; Raghavachari, K.; Rendell, A.; Burant, J. C.; Iyengar, S. S.; Tomasi, J.;

Cossi, M.; Rega, N.; Millam, N. J.; Klene, M.; Knox, J. E.; Cross, J. B.; Bakken, V.;

Adamo, C.; Jaramillo, J.; Gomperts, R.; Stratmann, R. E.; Yazyev, O.; Austin, A. J.;

Cammi, R.; Pomelli, C.; Ochterski, J. W.; Martin, R. L.; Morokuma, K.; Zakrzewski, V.

G.; Voth, G. A.; Salvador, P.; Dannenberg, J. J.; Dapprich, S.; Daniels, A. D.; Farkas, O.;

Foresman, J. B.; Ortiz, J. V.; Cioslowski, J.; Fox, D. J. *Gaussian 09, Revision D.01*;

Gaussian, Inc: Wallingford, CT, 2009.
